# Autonomy in adolescents in palliative care and its biopsychosocial impact: A systematic review

**DOI:** 10.1177/26323524251386501

**Published:** 2025-10-31

**Authors:** Ana Raquel Castro, Joana Brandão Silva, António Pereira Neves, João Rocha Neves, Hugo Ribeiro, Marília Dourado

**Affiliations:** 1 Faculty of Medicine, University of Coimbra, Portugal; 2 School of Medicine and Biomedical Sciences, University of Porto, Portugal; 3 Department of Biomedicine—Unit of Anatomy, Faculty of Medicine of University of Porto, Portugal; 4 Vascular Service, Local Health Unit S. João, Porto, Portugal; 5 RISE-Health, Faculty of Medicine, University of Porto, Portugal; 6 Community Palliative Care Team Gaia, Local Health Unit Gaia and Espinho, Vila Nova de Gaia, Portugal; 7 Coimbra Institute for Clinical and Biomedical Research, Portugal

**Keywords:** pediatric palliative care, adolescence, autonomy, clinical complexity, psychosocial support systems, empowerment in healthcare

## Abstract

**Background:**

Adolescence is a period of significant physical, psychological, and social changes, which can be intensified by the diagnosis of serious or chronic illnesses. This makes promoting autonomy in palliative care particularly challenging.

**Aim:**

The present review aimed to identify factors and strategies that promote the autonomy of adolescents in palliative care.

**Design:**

We conducted a systematic review following Preferred Reporting Items for Systematic Reviews and Meta-Analyses (PRISMA) guidelines, searching PubMed, Web of Science, and Scopus. This systematic review synthesized existing literature and evidence regarding communication, involvement, and psychosocial support strategies in adolescent palliative care.

**Methods:**

The study examined various tools and models, including Voicing My Choices, Building Our Solutions Together for Pediatric Advance Care Planning (BOOST-pACP), and the Family-Centered Advance Care Planning (FACE) model, to evaluate their effectiveness in facilitating communication, family support, and decision-making.

**Results:**

Effective communication tools and family-centered approaches are crucial for promoting adolescent autonomy. Strategies focused on open communication, psychosocial support, and active adolescent involvement in decisions can enhance autonomy, although further research is needed to assess their effectiveness.

**Conclusion:**

Implementing communication facilitation, psychosocial support, and adolescent involvement strategies is essential. Despite the need for more evidence, the review offers a set of recommendations to support the autonomy of adolescents in palliative care.

## Plain language summary

Supporting teen autonomy in palliative care: Key strategies and approaches

Adolescence is a time of big physical and emotional changes, which can be made even more challenging when teens have serious or long-term illnesses. It’s important to help these young people take part in decisions about their care, but this can be difficult. This review looked at ways to support teens’ independence and involvement in their own care. It found that good communication, involving teens in decisions, and providing emotional support are very helpful. Tools like Voicing My Choices and BOOST-pACP make it easier for teens, their families, and healthcare teams to work together. The family-centered FACE model also helps families connect and meet everyone’s needs. Using these strategies can help teens feel more in control and respected. More research is needed to see how well these approaches work, but for now, these strategies are recommended to support adolescents in palliative care.

## Introduction

Globally, there is an increasing recognition of the importance of addressing serious illnesses and enhancing the quality of life for individuals facing life-limiting conditions. Palliative care (PC), as defined by the World Health Organization (WHO), offers an integrated and multidisciplinary approach to meet these needs.^1^ PC aims to improve the quality of life for patients and their families through comprehensive symptom management and the provision of emotional, social, and spiritual support. Alleviating suffering throughout the illness journey is key, from diagnosis through mourning.^1,2^ While PC is a valuable resource for many, tailoring these approaches is crucial for specific populations, particularly within the pediatric age group.

Within the broader framework of PC, pediatric palliative care (PPC) addresses the distinct requirements of children and adolescents facing serious or terminal illnesses. In Portugal, PPC encompasses care for individuals from birth to 17 years and 364 days, as outlined in Order No. 9871/2010 of June 11th. A key challenge within PPC lies in mitigating the significant emotional impact these conditions have on young individuals. A premature death can threaten dreams and destroy goals.^3^ Adding to this complexity, the uncertainty of prognosis and the often-extended clinical course of disease in children can contribute to increased suffering and feelings of loss.^3,4^ Thus, providing PPC requires sensitivity to the developmental needs of children and adolescents.^4,5^

The WHO sees adolescence (10–19 years old) as a period of time of fast growth and big changes. A lot happens with their bodies, how they think, and their social lives. It is a period characterized by physical, psychological (such as identity formation), and social changes (such as the growing need for independence). In this transition between childhood and adulthood, young people face multiple challenges, such as adapting to body changes, peer pressure, exploring new relationships, and defining goals for the future, accompanied by a growing need for autonomy and personal affirmation.^6,7^ All these processes are very important during the decision of health, especially with adolescents with high clinical complexity due to advanced diseases and in need of PC.^6,7^

Autonomy is a fundamental and unavoidable ethical principle^8^ in PC. Recognizing that decision-making capacity evolves with age and maturity, it is essential to actively encourage and support patients in participating in decisions about their treatment—whether in setting goals, selecting therapeutic options, or expressing care preferences—while ensuring that their choices are respected and valued. This approach not only strengthens personal dignity,^9^ but also warrants that their voices are heard and respected in the therapeutic process,^10^ promoting a more patient-centered therapeutic relationship and the creation of relationships of trust, respect, and acceptance, which favor adherence to therapy.

Recognizing both the significance of autonomy and the complexities faced by adolescents with life-limiting illnesses,^11^ there is a clear need to identify factors and strategies that promote their autonomy within PC. This systematic review aims to address this gap by synthesizing existing literature on the biopsychosocial impact of autonomy and autonomy-promoting interventions in adolescent PC. The review will explore communication, involvement, and psychosocial support strategies. Ultimately, this review seeks to provide recommendations to support healthcare professionals and families in fostering adolescent autonomy and enhancing the quality of life for young people facing serious illness.

## Material and methods

This systematic review was conducted and reported in accordance with the Preferred Reporting Items for Systematic Reviews and Meta-Analyses (PRISMA) 2020 statement.^12^

To carry out this review, a bibliographic search was carried out in the PubMed, Web of Science, and Scopus databases on November 16, 2024. The selection of studies from the retrieved articles, based on our inclusion criteria, was completed by December 12, 2024. The search strategy incorporated MeSH terms and keywords, including: “*Palliative Care*,” “*Adolescent*,” “*Personal Autonomy*,” “*Decision Making*,” “*Barriers*,” “*Advanced Care Planning*,” and “*End-of-life care*.” The vocabulary was adapted for each database, ensuring the necessary coverage. There were no restrictions regarding the year of publication.

The complete search strategies used in each database are provided in Supplemental File 1.

### Inclusion and exclusion criteria

Studies were included if they met the following criteria: studies that address adolescent autonomy or decision-making, studies that involve adolescents (or young adults where the average age of the sample is less than 19 years old) in PC, studies that assess the impact of adolescents’ autonomy or decision-making on their quality of life, psychosocial well-being, or family dynamics, studies that are published as full-text articles, studies in English and Portuguese, studies that use qualitative, quantitative, or mixed-methods designs, studies that address experiences of adolescents in PC, focusing on autonomy, communication, and psychosocial support in different clinical contexts (cancer, human immunodeficiency virus (HIV), and cystic fibrosis), and studies that discuss advanced care planning (ACP) tools in playing an essential role in promoting adolescent autonomy by facilitating open and transparent end-of-life communication.

Studies were excluded if they met any of the following criteria: studies that focus on adult PC without any specific relevance to adolescents, studies that involve participants diagnosed with a psychiatric illness or without the cognitive capacity to understand the decision-making process, studies that do not explicitly address autonomy or decision-making by adolescents in PC, studies that do not evaluate the impact of autonomy on quality of life, abstracts of posters or other scientific presentations, letters, editorials, and research protocols, studies that focus solely on the experiences of healthcare providers or family members, without directly addressing the perspectives of adolescents, studies that do not provide sufficient information about the age of participants to determine if they meet the inclusion criteria, studies that focus solely on pharmacological interventions without considering the biopsychosocial aspects of autonomy, studies not available in full text and studies that do not have the population average age with less than 19 years old.

The inclusion criteria covered studies recognizing the variability in the definitions of adolescence and the overlap with the beginning of adulthood in some contexts; the inclusion of young adults up to 25 years old was allowed, as long as the average age of the study sample in question was less than 19 years old. We acknowledge that this decision could introduce selection bias, as the experiences of individuals in the 18–25 age range may differ from those of younger adolescents. To mitigate this, we carefully examined the characteristics and findings of studies that included participants over 19 to assess whether their inclusion significantly altered the overall findings. In our analysis, we found that the inclusion of these participants did not substantially change the overall patterns and themes related to adolescent autonomy.

After exporting the search results from all databases into Mendeley, we used Mendeley’s built-in duplicate detection function to identify and remove exact duplicates based on fields such as title, authors, and publication year. All potential duplicates identified by the software were then manually reviewed to ensure accuracy. After duplicate removal, two authors (A.C. and M.D.) independently participated in study selection; any disagreement was solved by the intervention of a third author (H.R.). Initially, studies were selected by title and abstract, and the remaining ones were eligible for full-text assessment. The selected studies were carefully revised to avoid including repeated populations. To identify and exclude studies reporting data from the same participant group, we carefully examined the authors, study settings (e.g., hospitals and clinics), and study timeframes of potentially overlapping studies. If the authors, setting, and timeframe were the same or highly similar, we investigated further to determine if the studies involved the same participants. If we determined that studies involved the same participant group, we included only the study with the most comprehensive data or the clearest reporting of the relevant outcomes.

Two authors (A.C. and M.D.) independently extracted data from included studies. Data were extracted using a purposely built form on the year of publication, country, and center of recruitment, study design, recruitment time, number of participants, participants’ age, gender distribution, and comorbidities.

Studies whose population consisted of participants diagnosed with a psychiatric illness or without the cognitive capacity to understand the decision-making process, regardless of the diagnosis, were excluded. In addition, studies that did not explicitly address autonomy or decision-making by adolescents in PC or end-of-life care were excluded, as well as studies that did not evaluate the impact of autonomy on quality of life. Abstracts of posters or other scientific presentations, letters or editorials and research protocols were also excluded.

The Strength of Recommendation Taxonomy (SORT) scale^48^ was used to assign levels of evidence and strengths of recommendation. The SORT scale is a patient-centered approach to grading evidence that considers the following factors: the quality of the research design, the consistency of the findings, the amount of patient-oriented evidence, and the clinical relevance of the outcomes. SORT assigns each study a grade of A, B, or C (A: indicates consistent and good-quality patient-oriented evidence; B: indicates inconsistent or limited-quality patient-oriented evidence; C: indicates consensus, usual practice, opinion, disease-oriented evidence, or case series (for studies of diagnosis, treatment, prevention, or screening)). We used these SORT grades to synthesize the evidence and formulate recommendations, giving greater weight to studies with higher SORT grades.

To address the study aims, a data extraction form was developed to capture relevant information from each included study. The form included sections on: (1) Factors influencing adolescent autonomy in PC (e.g., family dynamics, communication styles, cultural beliefs, healthcare provider attitudes, adolescent’s cognitive and emotional development); (2) Strategies for promoting adolescent autonomy (e.g., advance care planning, shared decision-making, communication skills training, psychosocial support programs); (3) Biopsychosocial impact of these factors and strategies (e.g., quality of life, psychological well-being, social functioning, and spiritual well-being); and (4) Study design and population characteristics (e.g., sample size, participant characteristics, and setting). This data extraction process allowed us to systematically gather information on the key elements related to our research question, enabling us to identify common themes, patterns, and gaps in the literature.

## Results

The bibliographic search identified 246 articles—92 articles in PubMed, 26 articles in Web of Science, and 128 articles in Scopus, with 70 duplicates excluded.

By reading the respective titles and abstracts, 169 articles were excluded, meaning that only 7 met the inclusion criteria. The full reading and detailed analysis of the articles were carried out by two authors. The third author carried out an audit when there was disagreement. This resulted in the exclusion of an article due to the lack of information regarding the average age of the sample.

**Figure 1.**
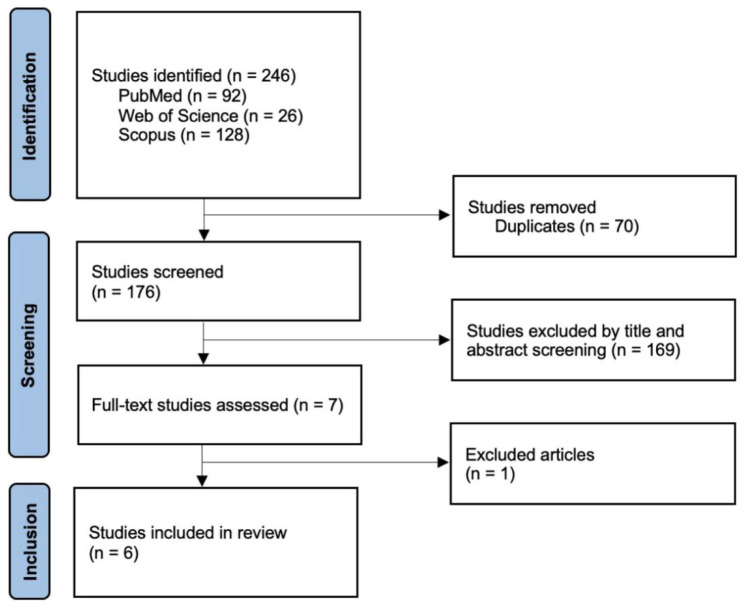
Study selection flowchart, according to the PRISMA 2020 methodology.^12^ PRISMA: Preferred Reporting Items for Systematic Reviews and Meta-Analyses.

Therefore, six articles were included in the present review, as shown in the research results flowchart, by the PRISMA 2020 standard^12^ (Figure 1). The articles were evaluated for methodological quality using the GRADE scale,^13^ with the results presented in Table 1.

**Table 1.** Adolescents in palliative care—perspectives, decisions, and support: synthesis of studies that explore the experiences, autonomy and psychosocial support of adolescents in palliative care, addressing clinical contexts such as cancer, HIV, cystic fibrosis, and end-of-life care.

Authors	Study design	Objective	Population and setting	Factors influencing autonomy	Key outcomes	SORT
Weaver et al.^14^	Qualitative study	Analyze how the roles of “being a good child” and “being a good patient” are experienced and influence care decisions	40 teenagers (12–19 years old), diagnosed with cancer, undergoing treatment Hospital inpatient unit	The concepts of “good patient” and “good son” are socially constructed by adolescents with cancer and positively and negatively influence their illness experiences, decision-making, and personal relationships	Understanding the social roles played by adolescents with cancer is essential for developing personalized and effective interventions that consider their social and emotional needs	3
Lyon et al.^15^	RCT	Test the effectiveness of the FACE^a^ model in advance care planning	38 family-adolescent dyads (14–21 years) with HIV Hospital outpatient setting	The FACE model contributed to a significant increase in congruence between adolescents and family members about end-of-life care preferences and a reduction in decision-making conflict. It demonstrated an improvement in the quality of communication between everyone involved	The FACE model is effective in increasing the congruence and quality of communication about end-of-life care for adolescents with HIV	1
Penson et al.^16^	Clinical case report	Explore the psychosocial challenges inherent to adolescence in a patient at the end of life and the impact of support, communication, and connection on decision-making	19-year-old young man diagnosed with leukemia, in the terminal stage Hospital inpatient unit	The ability to make decisions may vary over the course of the disease. Adapting expectations to this reality allows the teenager to get involved in the decision-making process in a more comfortable and effective way	Psychosocial support, a multidisciplinary approach and good communication allow the expression of fears, desires, and preferences, contributing to more active participation	3
Fairweather and Jones^17^	Meta-analysis of qualitative studies	Understand how empowering patients with cystic fibrosis influences their illness experience and identify strategies that promote their empowerment	Children and adolescents diagnosed with cystic fibrosis in pediatric palliative care Hospital outpatient setting	Training improves quality of life and adherence to treatments. Factors that promote it: positive interpersonal relationships, access to information and participation in decisions	Promoting social support, access to information, respect, opportunities for mastery, and competence is critical to empowering adolescents with cystic fibrosis and ensuring they can live fuller, more independent lives	1
Trang et al.^18^	Narrative review	Investigate the importance of including children and adolescents in discussions about ACP and identify the challenges and opportunities for this practice	Adolescents (13–19 years old) in palliative care Hospital outpatient setting	The PAC presents challenges related to the assessment of decision-making capacity, communication, and cultural issues. It is possible to overcome them through family support, clear communication, and the use of specific tools. The adolescent’s voice must be valued and their preferences considered	The PAC is essential to ensure that the desires and values of teenagers are respected. It is necessary to invest in strategies to facilitate these conversations, such as training health professionals and developing specific tools	2
Barton et al.^19^	Qualitative study	Understand the experiences, needs and perspectives of adolescents with cancer, focusing on psychosocial and emotional challenges	32 adolescents (14–25 years old), undergoing treatment for advanced cancer Hospital inpatient unit	Uncertainty about the future, the difficulty in making complex decisions about treatment, and social isolation were recurring themes in the interviews. Participants expressed the need for psychological and social support to deal with emotions and challenges related to the disease	There is a need for the development of services that integrate comprehensive psychosocial and mental health support to help navigate complex decisions, alleviate emotional burden, and provide more support	3

SORT: Strength of Recommendation Taxonomy; FACE: Family-Centered Advanced Care Planning model; HIV: human immunodeficiency virus; PAC: advanced care planning; RCT: randomized clinical trial.

The characteristics of the included studies are shown in Table 1.

The articles included analyzed the experiences of adolescents in PC, focusing on autonomy, communication, and psychosocial support in different clinical contexts, such as cancer, HIV, and cystic fibrosis.

In general, studies have shown that adolescent autonomy is influenced by social, emotional, and clinical factors. Open communication and emotional support were identified as essential elements to promote the active participation of adolescents in decisions related to care. It was also highlighted that autonomy can fluctuate according to the evolution of the disease, requiring adaptations in the support offered.

In particular, each study contributed important insights^14–19^:

- *Weaver et al.*^14^: Social pressure to play the roles of “good patient” and “good son” can significantly limit the autonomy of adolescents with cancer. This social expectation, internalized by young people, leads them to prioritize family needs and adherence to treatment, even if this conflicts with their own desires and needs. This dynamic can have a significant impact on the psychological well-being of adolescents and the quality of their care, highlighting the importance of interventions that promote the autonomy and empowerment of this young population.
- *Lyon et al.*^15^: The Family-Centered Advance Care Planning model (FACE) intervention is an effective tool for promoting the autonomy of adolescents with HIV in the PAC process, increasing congruence between the preferences of the adolescent and their family, and facilitating open communication about complex topics. This synergistic approach contributes to greater satisfaction of adolescents and their families with the care plan, in addition to reducing the stress and anxiety associated with making end-of-life decisions.
- *Penson et al.*^16^: The clinical case report illustrated the variability in the autonomy of a young woman with leukemia, who initially actively participated in decisions, but became more dependent as the disease progressed.
- Fairweather and Jones^17^: Empowerment of young people with cystic fibrosis is a multifactorial process, influenced by positive interpersonal relationships, access to accurate information and opportunities to make decisions about their care. By feeling supported, informed and able to influence their own lives, these young people develop a greater sense of autonomy and well-being.
- *Trang et al.*^18^: PAC tools play an essential role in promoting adolescent autonomy by facilitating open and transparent end-of-life communication and ensuring their voices are heard in decisions about their care.
- *Barton et al.*^19^: Adolescents with advanced cancer experience a series of difficulties, including uncertainty about the future, social isolation, and complex decision-making, highlighting the importance of adequate psychosocial support.

## Discussion

The studies analyzed in this review highlight several factors influencing patient-centered communication and adolescent decision-making in healthcare contexts. Overall, the findings indicate that communication plays a crucial role in fostering autonomy and strengthening the relationship between adolescents, families, and healthcare professionals.^14,15,17–19^ However, they also reveal significant challenges, such as the impact of family dynamics on adolescent participation^16^ and the difficulties professionals face in addressing sensitive topics effectively. While some studies suggest that family involvement supports the decision-making process,^15,18^ others point out that it may, in certain cases, limit adolescent agency, particularly when conflicting perspectives or reluctance to engage in end-of-life discussions arise.^14,16,19^

The heterogeneity of the included studies is an important factor to consider when interpreting these findings. Variations in methodology, study populations, and healthcare settings create challenges in drawing direct comparisons, underscoring the need for further research with more robust designs and representative samples. In addition, the lack of a standardized definition of concepts such as empowerment and decision-making capacity may limit the applicability of findings in clinical practice.^17,18^ Despite these limitations, the reviewed studies reinforce the importance of communication strategies that are sensitive to the individual needs of adolescents and their families, ensuring that their voices are actively considered in care planning.^14,15,19^

The patient-centered communication model requires a comprehensive understanding of their needs, values, and context.^20^ It aims to promote autonomy, trust, and adherence to health management.^21^ In pediatrics, the need for communication adapted to the particularities of this age group is unquestionable. Its effectiveness requires active listening, frequent and structured conversations, sensitivity and affection, and gradual transmission of information appropriate to their age and with parental consent.^22^

The findings of Weaver et al.^14^ are consistent with the literature in the sense that adolescents value open communication and the establishment of an honest relationship.^23,24^ This trust is crucial for adolescents to feel safe to share relevant information about their health, positively influencing the quality of care provided.^16,24,25^ The parental bond, based on deep knowledge of the adolescent’s values and preferences, positions parents as central defenders of their children’s rights, interests and beliefs. In this way, they are the ideal link between health professionals and the adolescent.^14^

Triadic communication, which involves adolescents, parents or caregivers, and health professionals^26^ is an interaction model that recognizes the importance of children and adolescents as active participants in conversations about their health. Therefore, the FACE model^15^ is a valuable tool for clinical practice: it establishes a guide for conversations about future care, actively includes the family and caregivers, and creates a safe environment that facilitates the expression of desires and concerns, making the decision-making process more harmonious, as corroborated by the congruence of family-adolescent dyads in studies by Lyon et al.^15^ and Needle et al.,^27^ who concluded that the knowledge remained valid for 1 year, suggesting its repetition annually.

On the contrary, family involvement can harm the adolescent’s active participation.^14,16,26^ The complexity of parental care intensifies in situations of illness, reaching a peak of difficulty and suffering in the face of a life-threatening condition. The emotional overload faced by families in PC^16,28,29^ destabilizes the family system, according to Bowen’s family systems theory (1978).^30^ This stress can lead to negative interactions, such as conflicts and dissatisfaction,^29^ and to maladaptive parenting practices that harm communication^28,31^—for example, unilateral decision-making, even if well-intentioned and driven by the fear of causing suffering to minors.^18,32,33^

Health decision-making capacity, defined as an adolescent’s ability to understand information, evaluate therapeutic options, and express an informed choice,^32–34^ depends not only on chronological age, but on their cognitive, emotional, and social capabilities.^32^ It results from the interaction of intrinsic factors, such as emotional and cognitive maturity, and extrinsic factors, including the quality of established communication,^33^ as the quality and way in which information is transmitted directly influences informed decision-making.^21,22,24,35^

The dynamic model that was proposed to evaluate this capacity highlights the importance of a multidisciplinary approach that recognizes the interdependence between these factors and contextualizes them in the adolescent’s psychological development.^33,36^

Previous experience in disease setting also contributes to the development of decision-making competence. The practice of self-management in chronic diseases, such as cystic fibrosis^17^ or type 1 diabetes mellitus,^37^ strengthens confidence and promotes active participation. There is consensus on the importance of nurses in education and teaching self-care.^16,18,38^

Adolescents in PC face profound challenges, including loss of autonomy and social isolation.^3^ Interventions such as Dignity Therapy and Meaning-Centered Psychotherapy help adolescents find meaning in adversity, promoting dignity, and emotional well-being.^39,40^ Reflection on personal values allows for a positive reinterpretation of the experience, reducing anxiety and depression. The “path of resilience” suggests the acceptance and exploration of meanings as central steps to dealing with death through a good life experience.^39^

Participation in group activities with peers offers emotional support and combats isolation, creating a space for exchanging experiences and strengthening identity. Digital platforms expand the reach of these initiatives, enabling continuous connection even under restrictive conditions.^41^

The PAC mentioned in international literature can be equated to the Individual and Integrated Care Planning,^42^ developed in Portugal. These are tools that promote a holistic, structured, and personalized approach to care.

The pediatric ACP (PACp)^43,44^ emerged through the adaptation of the PAC to pediatric particularities, motivated by the increasing complexity of pediatric care, the increased longevity of children with complex health conditions, and the greater recognition of the need for child- and family-centered approaches, while supporting healthcare professionals in addressing difficult but essential discussions. The PACp process uses specific tools, such as the FACE,^15^ Voicing My Choices,^45^ and BOOST-pACP (Building Our Solutions Together for Pediatric Advanced Care Planning)^46^ models. Other tools can be found in the literature, appropriate to various chronic disease contexts and age groups.^47^

Voicing My Choices is an approach based on open-ended questions that promotes reflection and is adaptable to the individuality and values of each adolescent.^45^

BOOST-pACP^46^ is a recent and improved tool, aimed especially at cancer patients between 10 and 18 years of age. It consists of a systematic and personalized approach, which includes components such as manuals, structured conversation sessions using flashcards, preparation materials that can be sent home, educational videos, and a summary sheet completed together by the family. In practice, this model not only facilitates a safe environment for discussions about care but also promotes open communication and improves congruence between clinical decisions and the values of the adolescent and their family.^43,44,46–48^

The PIIC, implemented in Portugal with a focus on adults, recognizes the existence of complex contexts, such as pediatric care.^42^ It is a tool that aims to personalize and coordinate care, respecting the clinical, psychological, and social needs of each patient. This personalized approach facilitates the integration of multidisciplinary care, promoting informed, patient-centered decisions.^42^

### Limitations

This review has several limitations that should be considered when interpreting the findings. First, we acknowledge that our search strategy was limited to three databases and did not include manual searching of Google Scholar or backward/forward citation chasing, which may have resulted in some publication bias. While these databases are highly regarded and cover a wide range of publications, it is possible that some relevant studies, particularly those not published in traditional academic journals or indexed in these databases, were not identified. Second, we included studies with varying levels of methodological rigor, as assessed by the SORT criteria. While we prioritized including studies with high-quality evidence, we also made a deliberate decision to include some studies with lower levels of evidence (e.g., case reports and studies with small sample sizes). We included these studies because they provided valuable and unique insights into the experiences of adolescents in PC and the practical strategies used to promote their autonomy and well-being. However, it is important to acknowledge that the findings from these studies may be less reliable and generalizable than those from studies with stronger designs. Therefore, our findings should be interpreted with caution, and future research should focus on rigorously evaluating the effectiveness of these strategies using more robust study designs.

Although this study offers insights into adolescent autonomy within PC, it is crucial to acknowledge the scarcity of research explicitly examining the impact of cultural factors. Variability exists across cultures concerning the adolescent’s role within the family, decision-making power, and communication styles related to health. To enhance the relevance and effectiveness of PC for adolescents from diverse backgrounds, future research should incorporate culturally sensitive methodologies. This includes qualitative studies to understand culturally specific perspectives on autonomy, family dynamics, and communication, as well as the adaptation and testing of interventions to ensure their appropriateness and acceptability across different cultural contexts. Acknowledging and addressing these cultural factors are crucial to promoting truly patient-centered care for adolescents in palliative settings.

## Conclusion

This article highlights the importance of tailored strategies to enhance autonomy and quality of life for adolescents in PC.

Adapting models such as the Integrated Individual Care Planning (PIICp) to pediatrics is crucial for offering patient-centered, ethical, and evidence-based care.

Key recommendations include involving adolescents in decision-making according to their cognitive and emotional development, using tools such as Voicing My Choices and BOOSTpACP to express preferences, and enabling gradual responsibility increases through targeted education.

The plan should be flexible, with periodic reviews adapting to health and maturity changes. Family involvement and psychosocial support—such as Dignity Therapy and peer groups—are vital for holistic care.

Training healthcare professionals in respectful communication and maintaining organized documentation are essential components.

The article emphasizes ongoing research and professional development to promote active adolescent participation, ensuring care aligns with individual values, needs, and aspirations throughout their palliative journey.

## Supplemental Material

sj-docx-1-pcr-10.1177_26323524251386501 – Supplemental material for Autonomy in adolescents in palliative care and its biopsychosocial impact: A systematic review Supplemental material, sj-docx-1-pcr-10.1177_26323524251386501 for Autonomy in adolescents in palliative care and its biopsychosocial impact: A systematic review by Ana Raquel Castro, Joana Brandão Silva, António Pereira Neves, João Rocha Neves, Hugo Ribeiro and Marília Dourado in Palliative Care and Social Practice

sj-docx-2-pcr-10.1177_26323524251386501 – Supplemental material for Autonomy in adolescents in palliative care and its biopsychosocial impact: A systematic review Supplemental material, sj-docx-2-pcr-10.1177_26323524251386501 for Autonomy in adolescents in palliative care and its biopsychosocial impact: A systematic review by Ana Raquel Castro, Joana Brandão Silva, António Pereira Neves, João Rocha Neves, Hugo Ribeiro and Marília Dourado in Palliative Care and Social Practice
