# Supplementary material for: Autonomy in adolescents in palliative care and its biopsychosocial impact: A systematic review
Source: Palliat Care Soc Pract. 2025 Oct 31;19:26323524251386501. doi: 10.1177/26323524251386501 (PMC12579122; doi:10.1177/26323524251386501)
Supplement: sj-docx-2-pcr-10.1177_26323524251386501 – Supplemental material for Autonomy in adolescents in palliative care and its biopsychosocial impact: A systematic review [file sj-docx-2-pcr-10.1177_26323524251386501.docx]

**Autonomy in adolescents in palliative care and its biopsychosocial impact: a systematic review**

**Supplementary File 1.**

In the PubMed database, the following search equation was used: ("Palliative Care"[MeSH] OR "Palliative Care"[All Fields] OR "Pediatric Palliative Care"[All Fields] OR "End-of-life care"[All Fields]) AND ("Adolescent"[MeSH] OR "Adolescent"[All Fields] OR "Teenagers"[All Fields] OR "Youth"[All Fields]) AND ("Personal Autonomy"[MeSH] OR "Autonomy"[All Fields] OR "Self-determination"[All Fields] OR "Decision Making"[MeSH] OR "Decision Making"[All Fields]) AND ("Barriers"[All Fields] OR "Challenges"[All Fields] OR "Obstacles"[All Fields] OR "Improvements"[All Fields]).

In the Web of Science database, the following search equation was used: TS=("Palliative Care" OR "Pediatric Palliative Care" OR "End-of-life care") AND TS=("Adolescent" OR "Teenagers" OR "Youth") AND TS=("Personal Autonomy" OR "Self-determination" OR "Decision Making") AND TS=("Barriers" OR "Challenges" OR "Obstacles" OR "Improvements" OR "Facilitators").

In the Scopus database, the following research equation was used: TITLE-ABS-KEY ( "Palliative Care" OR "Pediatric Palliative Care" OR "End-of-life care" ) AND TITLE-ABS-KEY ( "Adolescent" OR "Teenagers" OR "Youth" ) AND TITLE-ABS-KEY ( "Personal Autonomy" OR "Self-determination" OR "Decision Making" ) AND TITLE-ABS-KEY ( "Barriers" OR "Challenges" OR "Obstacles" OR "Improvements" OR "Facilitators" ).
